# Retraction: Investigation of the effect of UV-B light on *Arabidopsis* MYB4 (AtMYB4) transcription factor stability and detection of a putative MYB4-binding motif in the promoter proximal region of *AtMYB4*

**DOI:** 10.1371/journal.pone.0357636

**Published:** 2026-09-08

**Authors:** The PLOS One Editors

After this article [1] was published, concerns were raised regarding Figs 1 and S1. Specifically:

- Lanes 1–6 of the anti-MYB4 IgG panel in Figs 1A and 1B appear more similar to each other than would be expected from independent experiments when the levels and scale of each band/lane are adjusted.
- Lanes 3–5 of the anti-MYB4 IgG panel in Fig 1C appear similar to lanes 2–4 of Fig 3Q of a later publication by an overlapping author group [2], retracted in [3].
- There appears to be a vertical discontinuity between the Non-induced and Induced lanes of Fig S1C.

The first and corresponding authors responded to the above concerns and stated that the original, underlying images for this article are no longer available. The *PLOS One* Editors consider that the responses received do not resolve the concerns raised, and in the absence of the underlying image data, the above concerns cannot be fully resolved.

The corresponding author offered to repeat the experiments, but PLOS does not consider that repeat experiments alone are sufficient to resolve the concerns with the figures as published.

In light of the above concerns which call into question the reliability and integrity of the results in [1], the *PLOS One* Editors retract this article.

MM, PA, AK, and SR did not agree with the retraction. VB either did not respond directly or could not be reached
